# Giant enhancement of second harmonic generation from monolayer 2D materials placed on photonic moiré superlattice

**DOI:** 10.1515/nanoph-2023-0124

**Published:** 2023-10-10

**Authors:** Tingyin Ning, Lina Zhao, Yanyan Huo, Yangjian Cai, Yingying Ren

**Affiliations:** Shandong Provincial Engineering and Technical Center of Light Manipulations & Shandong Provincial Key Laboratory of Optics and Photonic Device, School of Physics and Electronics, Shandong Normal University, Jinan 250358, China

**Keywords:** second harmonic generation, moiré superlattice, 2D-material

## Abstract

We numerically investigate second harmonic generation (SHG) from a monolayer of 2D-material placed on photonic moiré superlattice fabricated by dielectric materials. The greatly enhanced local field at the resonance modes of moiré superlattice can dramatically boost the SHG response in 2D materials. Considering a typical 2D-material MoS_2_ monolayer placed on a photonic moiré superlattice of a twist angle 9.43°, the maximum SHG conversion efficiency reaches up to 10^−1^ at a relatively low intensity of fundamental light 1 kW/cm^2^, which is around 14 orders of magnitude larger than that from the monolayer placed on a flat dielectric slab without moiré superlattices. The SHG conversion efficiency from the monolayer can be further enhanced with the decrease of the twist angles of moiré superlattice due to the even more confinement of local field. The flat bands in the moiré superlattices formed by the small twist angles can particularly ensure the efficiency even under wide-angle illuminations. The results indicate that photonic moiré superlattice which can tightly confine light is a promising platform for efficient nonlinear optics.

## 1 Introduction

Second harmonic generation (SHG), as one of the most fundamental nonlinear optical phenomena, has important applications in the fields such as short-wavelength light sources, imaging, and sensing [1]. SHG in two-dimensional (2D) monolayer materials has received much interest and has been extensively investigated in recent years, since 2D materials, such as graphene and transition metal dichalcogenides (TMDs), possess large and tunable nonlinear susceptibilities in a broad band [2–7]. Such 2D materials can also be easily integrated into photonic platforms including fibers, waveguides, micro-rings, and so on, due to their excellent flexibility and mechanical properties [8–10]. However, the monolayer thickness unavoidably limits the efficiency of their nonlinear response. The boost of SHG in 2D materials is essentially required for applications. Until now, various methods have been utilized to enhance the nonlinear response in 2D monolayer materials, such as the excitation of surface plasmon [11–13], exciton resonance [14], 2D materials integrated with metallic substrate or nanostructures [15–17], photonic crystals [18–20], waveguide [21–23], or other cavities [24–26], etc.

The moiré superlattices, formed by two periodical patterns with a twist angle or a mismatched lattice constant, has drawn much attention due to the unexpected novel physical properties [27–31]. Besides the widely studied novel electronic states in condensed matter physics, the photonic moiré superlattices were found to have the ability to extremely confine the light field [32, 33]. Particularly, moiré superlattices possess flat bands in the whole moiré Brillouin zone. Flat bands have non-dispersive features which mean that the group velocity of light is zero to enhance the light–matter interaction. Further, the non-dispersive feature indicates that the resonant frequencies of moiré superlattices are independent on the angle of incidence. The light from different propagation directions, for example, when the light is focused on the nanostructure using objective lens of high numerical aperture, can work together to improve the efficiency in various applications. The other cavities, such as traditional photonic crystals of simple lattice, metasurfaces of (quasi-)BICs, can also have ultrahigh *Q*-factor and confine the field tightly at the resonance modes. However, there are no or just local flat bands, and the resonant frequencies highly depend on the propagation directions, which limits their performance under wide-angle illuminations. Thus, the photonic moiré superlattices have important applications in light–matter interactions, including low-threshold lasing [34], ultra-low level all-optical switching [35], wide-angle linear and nonlinear optical devices [36, 37], and tunable harmonic generations in the twisted 2D materials [38–40]. Just recently, the photonic moiré superlattice was demonstrated to boost SHG in monolayer van der Waals crystals [41]. The SHG efficiency in WS_2_ monolayer placed between the moiré superlattice was enhanced up to be 2.67 × 10^−8^ at the intensity of fundamental light 0.1 GW/cm^2^, which was 600 times larger than that from a free-standing WS_2_ monolayer. However, the photonic moiré superlattice used in the article, which was formed by a mismatched bilayer grating, with a 2D material monolayer between the stacks was not easy to be fabricated experimentally. Furthermore, the 2D material monolayers of nonzero absorption coefficient inside the cavity dramatically attenuate the quality factor of resonance systems.

In this article, we report the efficient SHG in monolayer 2D materials covered on a photonic moiré superlattice. The dramatically enhanced local field on the surface of photonic moiré superlattices leads to the efficient SHG at relatively low fundamental intensities. The flat bands in the photonic moiré superlattices formed by the small twist angles can particularly ensure the efficiency even under wide-angle illuminations. The results indicate the promising applications of photonic moiré superlattices in nonlinear optical harmonic generations.

## 2 Moiré superlattice and numerical method

**Figure 1:**
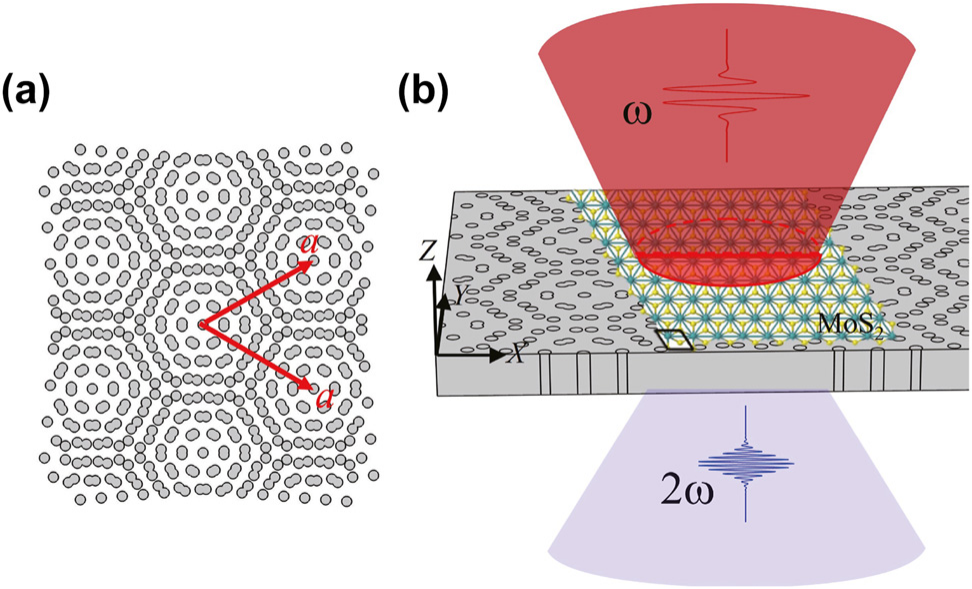
Schematic structure of (a) a moiré superlattice (*a* is the lattice constant, twist angle *θ* = 9.43°), and (b) a MoS_2_ monolayer placed on the moiré superlattice.

We consider a moiré superlattice formed by twisting two layers of triangular lattices in the same dielectric membrane, as investigated in Ref. [42]. Such moiré superlattices have been fabricated using the electron-beam lithography technique combining with the etching processing [34], indicating a feasible structure for experiments. Different twist angles *θ* will lead to the superlattices of different periods by *a* = *a*_0_/(2sin(*θ*/2)), where *a*_0_ and *a* are the lattice constants of basic triangular lattice and moiré superlattice, respectively. For example, when a twist angle is 9.43°, the lattice constant of the superlattice is around 3.04 μm. The schematic structure of a moiré superlattice of a twist angle 9.43° is shown in Figure 1(a). The lattice constant of the basic triangular lattice is 500 nm, and the radius of nanohole is 120 nm. Here, MoS_2_ monolayer, as typical TMDs, is considered. A MoS_2_ monolayer is placed on the surface of moiré superlattices, as shown in Figure 1(b).

The wave equation for the fundamental and SHG fields in the frequency domain (time-harmonic factor exp(−i*ωt*)) can be expressed as [1, 42],

(1) $$\nabla \times \nabla \times E\left(\omega \right)-k_{0}^{2}E\left(\omega \right)=\;\mu_{0}\omega^{2}P^{\left(1\right)}\left(\omega \right)$$

(2) $$\nabla \times \nabla \times E\left(\Omega \right)-\varepsilon_{2}k_{2}^{2}E\left(\Omega \right)=\;\mu_{0}\Omega^{2}P^{\left(2\right)}\left(\Omega \right)$$

where Ω = 2*ω* is the angular frequency of SHG, **E**(*ω*) and **E**(Ω) are fundamental and SHG electric fields, respectively. *k*_0_ = *ω*/*c* and *k*_2_ = Ω/*c* are the wave vectors of the fundamental and SH lights, respectively, where *c* is the speed of light in a vacuum. *ɛ*_0_ is the vacuum permeability. *ɛ*_2_ is the relative permittivity of materials at SH frequency.

The linear polarization for MoS_2_ and the dielectric moiré superlattice is written as **P** ^(1)^(*ω*) = *ɛ*_0_(*ɛ* − 1)**E**(*ω*), where *ɛ*_0_ and *ɛ* are the vacuum permittivity and the relative permittivity of materials, respectively. The *ɛ* of the dielectric materials is taken as 11.56, which corresponds to the materials, such as amorphous Al doped GaAs (Al_*x*_Ga_1−*x*_As) or silicon, and the *ɛ* of MoS_2_ is referred the experimental data [43]. Due to the symmetry structure of amorphous Al doped GaAs or Si, there is no bulk SHG response from the moiré superlattices. SHG from MoS_2_ is solely considered. For MoS_2_, the bulk crystal belongs to the *D*_6*h*_ space group and has the inversion symmetry, while the inversion symmetry is broken in the monolayer and it changes to be *D*_3*h*_ space group [44]. The non-vanished second-order nonlinear susceptibility of the MoS_2_ monolayer is $\chi^{\left(2\right)}\equiv \chi_{\mathrm{xxx}}^{\left(2\right)}=-\chi_{\mathrm{xyy}}^{\left(2\right)}=-\chi_{\mathrm{yxy}}^{\left(2\right)}=-\chi_{\mathrm{yyx}}^{\left(2\right)}$ [44]. The value of *χ* ^(2)^ of the MoS_2_ monolayer is around 45 pm/V at the wavelength region we concern in the article [7]. The components of **P** ^(1)^(*ω*) originating from the mixing of fundamental and SHG waves in MoS_2_ can be written as,

(3) $$P_{x}^{\left(1\right)}\left(\omega \right)=\varepsilon_{0}\chi_{\mathrm{xxx}}^{\left(2\right)}E_{x}^{*}\left(\omega \right)E_{x}\left(\Omega \right)-\varepsilon_{0}\chi_{\mathrm{xyy}}^{\left(2\right)}E_{y}^{*}\left(\omega \right)E_{y}\left(\Omega \right)$$

(4) $$P_{y}^{\left(2\right)}\left(\omega \right)=-\varepsilon_{0}\chi_{\mathrm{yxy}}^{\left(2\right)}E_{x}^{*}\left(\omega \right)E_{y}\left(\Omega \right)-\varepsilon_{0}\chi_{\mathrm{yyx}}^{\left(2\right)}E_{y}^{*}\left(\omega \right)E_{x}\left(\Omega \right)$$

and the components of **P** ^(2)^(Ω) in MoS_2_ is expressed as,

(5) $$P_{x}^{\left(2\right)}\left(\Omega \right)=\varepsilon_{0}\chi_{\mathrm{xxx}}^{\left(2\right)}E_{x}\left(\omega \right)E_{x}\left(\omega \right)-\varepsilon_{0}\chi_{\mathrm{xyy}}^{\left(2\right)}E_{y}\left(\omega \right)E_{y}\left(\omega \right)$$

(6) $$P_{y}^{\left(2\right)}\left(\Omega \right)=-2\varepsilon_{0}\chi_{\mathrm{yxy}}^{\left(2\right)}E_{x}\left(\omega \right)E_{y}\left(\omega \right)$$

The polarization in the MoS_2_ monolayer can also be described by the surface current using **J**(*ω*) = −i*ω* **P**(*ω*) or **J**(Ω) = −iΩ**P**(Ω) at the fundamental and SHG fields, respectively. In nonlinear surface current treatment, the bulk second-order nonlinear susceptibility *χ* ^(2)^ should be replaced by the surface second-order nonlinear susceptibility $\chi_{\text{S}}^{\left(2\right)}$ using $\chi_{\text{S}}^{\left(2\right)}=\chi^{\left(2\right)}d$ , where *d* is the effective thickness of the MoS_2_ monolayer [45]. Here, the thickness of MoS_2_ monolayer *d* = 0.62 nm is used.

**Figure 2:**
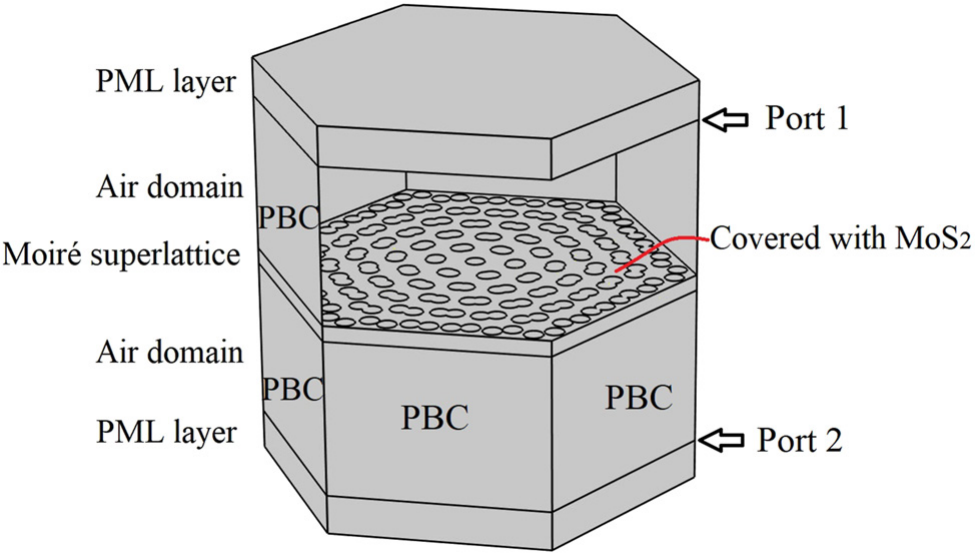
The simulation domain and related settings in the Comsol Multiphysics. The unit cell of moiré superlattice covered with a MoS_2_ monolayer is placed in the vacuum environment. The perfect matched layers (PML) are vacuum, and the periodic boundary conditions (PBCs) are employed on the surrounding boundaries.

The coupled equations can be numerically solved by finite element methods assisted by the commercial software Comsol Multiphysics in the frequency domain. The settings can be referred in our previous articles, such as [42]. The settings can be referred in our previous articles, such as [42]. Basically, the simulation domain of a unit cell of moiré superlattice is shown in Figure 2. For the linear optical properties, the conventional one step method is used. The top boundary is set as port 1 used for the incidence of light, and the component of electric field *E*_*x*_ or *E*_*y*_ corresponds to the *x*- or *y*-polarized illumination, respectively. The bottom boundary is noted as port 2. The periodic boundary conditions (PBCs) are employed on the surrounding boundaries, and the Floquet periodicity vector *k*_F_ is set as 0 at normal incidence. The transmission is calculated using |*S*_21_|^2^, where *S*_21_ is the *S*-parameter for the transmitted wave. The distribution of fields can be required at the desired wavelength. For the SHG simulation, two steps must be conducted. The first step is similar as the aforementioned settings to calculate the fundamental field which is used to express the second-order polarizations (Eqs. (5) and (6)). The second step is to calculate the generated second harmonic signal. The scatter field calculation with the zero background field is used. The PBCs are still employed on the surrounding boundaries, but the Floquet periodicity vector should be twice of fundamental *k*_F_ generally. The power of generated SHG signal, PSHG, can be obtained by the integration of Poynting flow at 2*ω* on the port one for reflected SHG and the port two for transmitted SHG. The conversion efficiency of SHG is defined as *η* = *P*_SHG_/*P*_in_, where *P*_in_ is the incident fundamental power. It is noting that for the monolayer MoS_2_, the surface current given by the fundamental and second-order polarizations is used to replace the real thickness layer.

The eigenfrequency and band structures are simulated by the eigenfrequency solver in Comsol Multiphysics. The settings in the domain of a unit cell of moiré superlattice are similar as those for linear optical properties. The Floquet vector *k*_F_ changes along the edges of irreducible Brillouin zone.

## 3 Results and discussion

**Figure 3:**
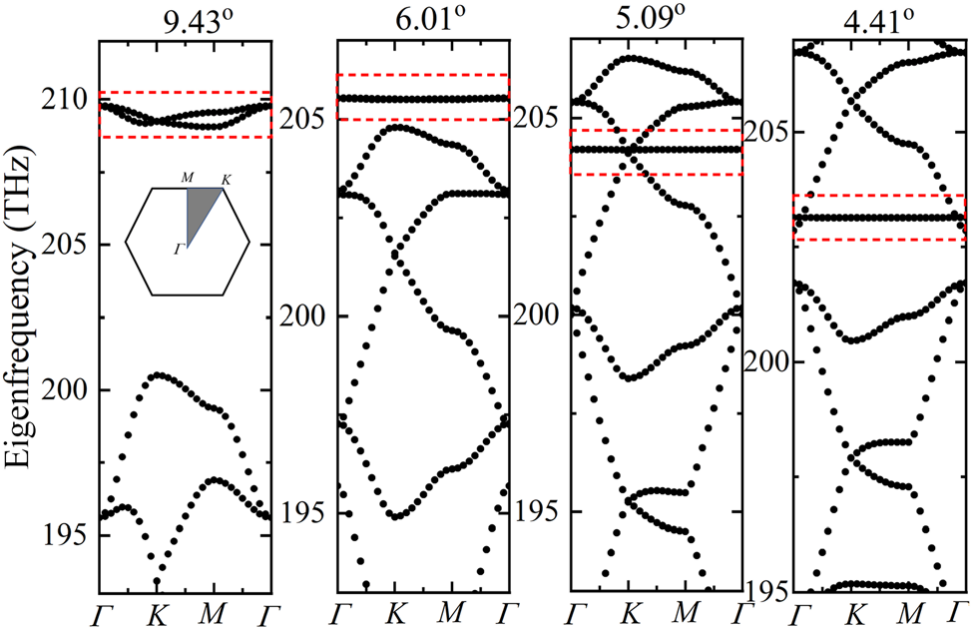
Band structures of moiré superlattices formed by different twist angles. The flat bands formed by the dipole modes *P*_*x*_ and *P*_*y*_ are marked in dashed red box. The inset of the first figure shows the Brillouin zone.

**Figure 4:**
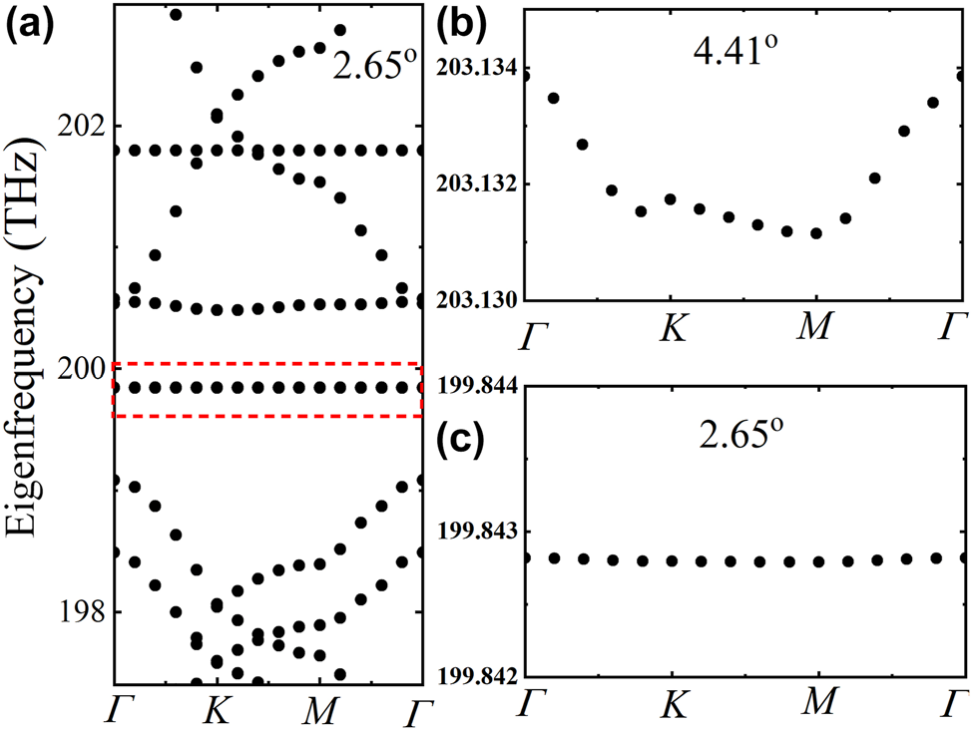
Band structure of moiré superlattices formed by the twist angle 2.65° and the flat band diagrams. (a) The band structure in a wide frequency range. (b) and (c) Are the enlarged band diagrams of flat band from the *p_x_* mode (marked in dashed red box) in moiré superlattices of the twist angle 4.41° and 2.65°, respectively.

**Figure 5:**
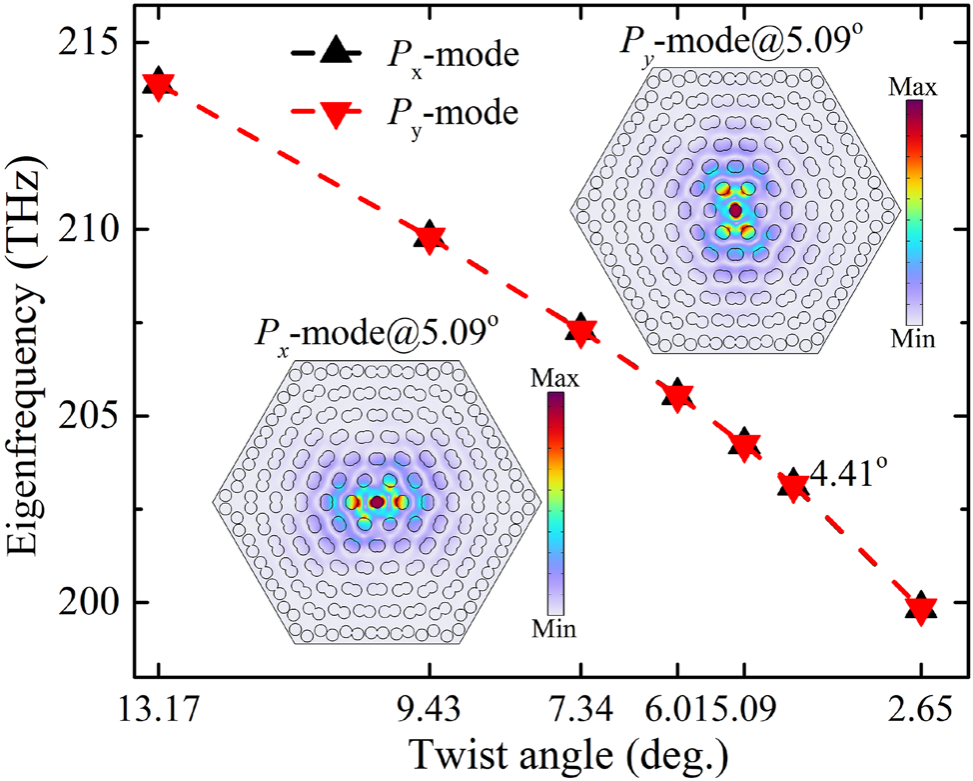
Eigenfrequency analysis of dipole modes *P*_*x*_ and *P*_*y*_ in moiré superlattices formed by different commensurate twist angles. The typical field distribution at *P*_*x*_ and *P*_*y*_ are shown in the inset of figure.

The eigenfrequency of the bare moiré superlattices is first studied to find the resonance modes. The band structures of moiré superlattices formed by different twist angles are shown in Figures 3 and 4. The dipole modes *P*_*x*_ and *P*_*y*_, which contribute to the flat bands are marked in dashed red box. Especially, the perfect flat band can be formed in the whole moiré Brillouin zone under the small angle 2.65° (Figure 4(b) and (c)) and even smaller angles. At such moiré superlattices, the non-dispersion flat bands are exactly immune to the light propagation directions. The nearly degenerated dipole modes *P*_*x*_ and *P*_*y*_ in moiré superlattices of different commensurate twist angles are specially shown in Figure 5. The field distributions of the two modes are shown in the inset of Figure 5. It is clear that the eigenfrequency can be significantly modulated by the twist angles, which can cover a broadband to enhance the nonlinear response of the 2D monolayer materials on the structures. Considering the computational consumption, we next mainly study the optical responses from the moiré superlattice of the twist angle 9.43° to demonstrate how the confined local field on the moiré superlattice to enhance the 2D monolayer MoS_2_ covered on it.

**Figure 6:**
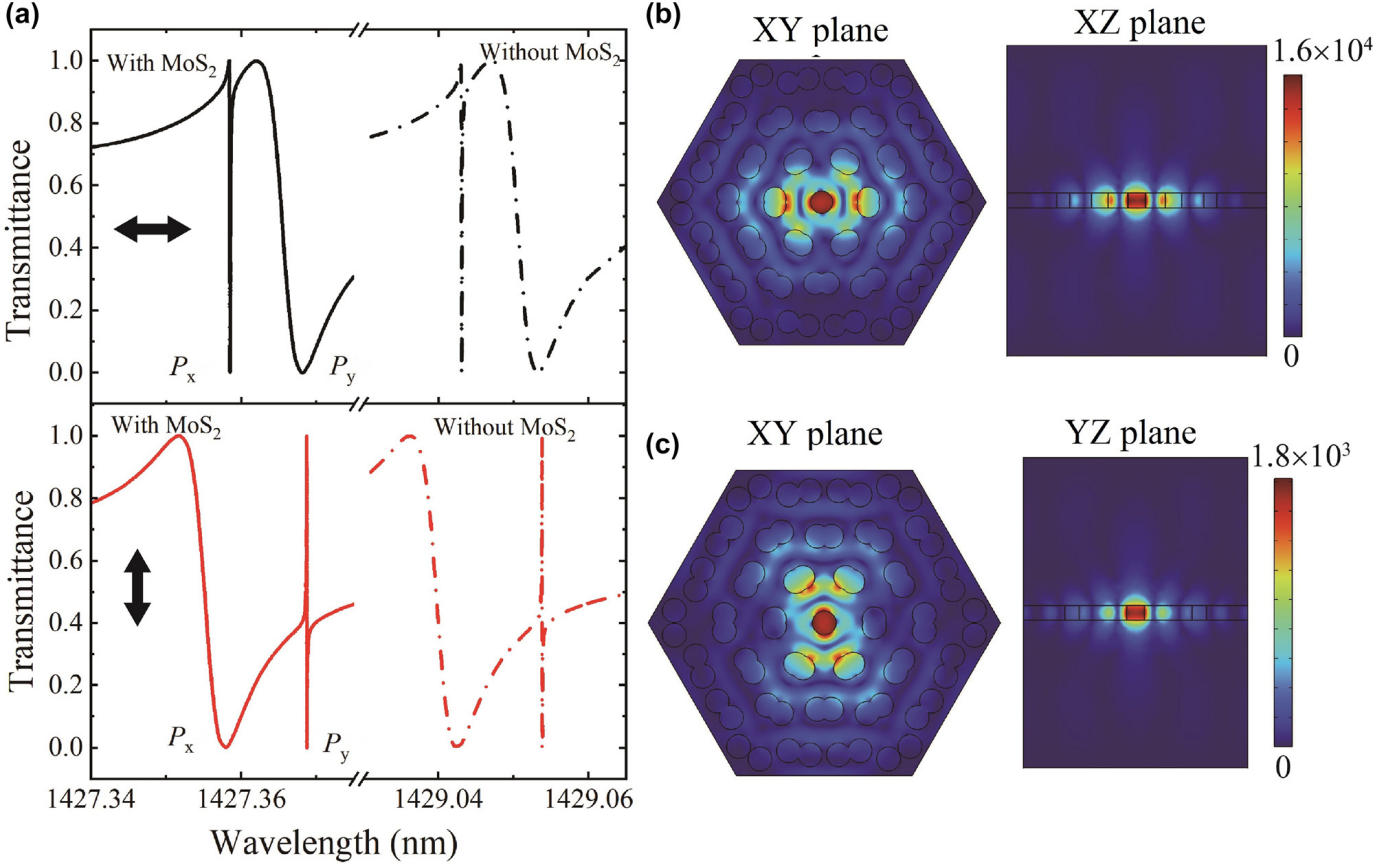
Linear transmittance and field distributions. (a) The linear transmittance of the nanostructure under the *x*- and *y*-polarized incidence, respectively. The bold arrows represent the directions of the polarization, along *x*- or *y*-axis. (b) and (c) Show the field distribution at *P_x_* and *P_y_* modes under the *x*-polarized incidence, respectively. (b) *XY*-plane and *XZ*-plane, and (c) *XY*-plane and *YZ*-plane. The values of colorbar represent the local field enhancement.

The linear transmittance in the nanostructure can be obtained by solving Eq. (1) when the SHG process is not considered, as shown in Figure 6(a). The light of the polarization along *x*- and *y*-axis at normal incidence is considered, respectively. The two dipole modes, *P*_*x*_ and *P*_*y*_, are observed, as those in the pure dielectric moiré superlattice [34, 35]. The resonance wavelength at *P*_*x*_ and *P*_*y*_ modes is around 1427.3585 nm and 1427.3685 nm, respectively. Comparing with the moiré superlattice without the MoS_2_ monolayer, the transmission spectra only have a blueshift due to the change of environment dielectric constant from air to the MoS_2_ monolayer. The lineshapes are almost kept since the MoS_2_ monolayer at such wavelength region has no absorption. The *Q* factors at *P*_*x*_ and *P*_*y*_ modes are calculated by *Q* = *λ*_r_/*δλ*_r_ for the Fano lineshape, where *λ*_r_ and *δλ*_r_ are the resonance wavelength and the difference of wavelength at peak and dip, respectively. The *Q* factors under the *x*(*y*)-polarization incidence are around 1 × 10^7^ (2.4 × 10^5^) and 2.4 × 10^5^ (6.5 × 10^7^) at the *P*_*x*_ and *P*_*y*_ modes, respectively. It is worth noting that the *Q* factor can also be obtained by the eigen-frequency solver. The values of the in-plane *Q* factor and vertical *Q* factor are infinity and 1.42 × 10^4^, respectively. So the incident light of the linear *x*- or *y*-polarization can excite the in-plane resonant modes but with out-of-plane decay or leaky modes to free space. The difference of the maximum *Q* factors at the incident light of *x*- and *y*-polarization may be ascribed to the different confinement size in a unit cell along *x*- and *y*-axis.

**Figure 7:**
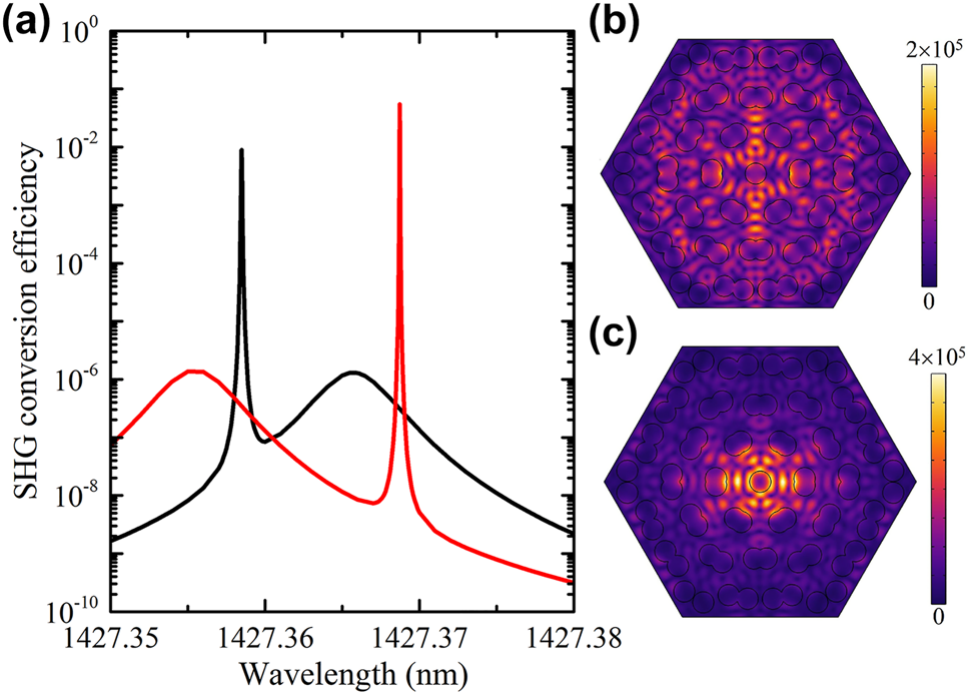
SHG response and field distributions. (a) Dependence of SHG conversion efficiency on the fundamental wavelength. The intensity of fundamental light is 1 kW/cm^2^. (b) and (c) Show the SHG field distribution in the *XY*-plane around the *P_x_* (fundamental light of the *x*-polarization) and *P_y_* (fundamental light of the *y*-polarization) resonance mode, respectively. The plane is chosen at the center of moiré superlattice. The units of colorbar are V/m.

**Figure 8:**
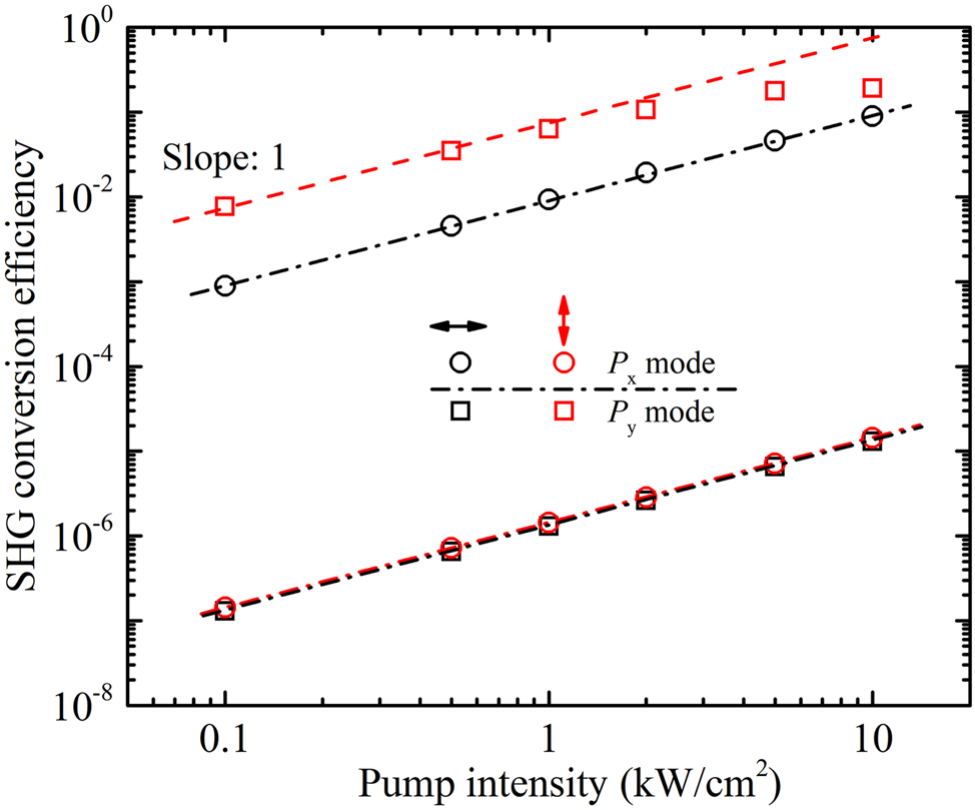
SHG conversion efficiency versus the input intensity of fundamental light at the *P*_*x*_ and *P*_*y*_ resonance mode when the polarization of fundamental light is along the *x*- or *y*-axis, respectively.

When the intensity of fundamental light is 1 kW/cm^2^, the spectra of SHG conversion efficiency under the fundamental light of different polarizations are shown in Figure 7(a). Two peaks appear around the resonance modes at the fundamental light of *x*- or *y*-polarization, respectively, due to the enhancement of local fundamental electric fields. The positions of the peaks of SHG conversion efficiency are not exact overlapped with the corresponding *P*_*y*_ and *P*_*x*_ resonance modes, since the SHG conversion efficiency is conjoint determined by the local fundamental field and the SHG field, and the **P** ^(1)^(*ω*) from the mixing of fundamental and SHG waves in MoS_2_ is not negligible to shift the resonance modes at such pump intensity. It is worth noting that maximum SHG conversion efficiencies arrive up to 10^−6^ and 10^−2^ around the *P*_*y*_ and *P*_*x*_ modes at the *x*-polarized fundamental light, respectively. The value even reaches up to about 10^−1^ around the *P*_*y*_ mode at the *y*-polarized fundamental light. For the MoS_2_ monolayer on the flat dielectric slab without moiré nanostructures, the conversion efficiency is only around 7.3 × 10^−17^ at the pump intensity 1 kW/cm^2^. The maximum enhancement of SHG is around 14 to 15 orders of magnitude at the *P*_*x*_ mode (*x*-polarized fundamental light) and *P*_*y*_ mode (*y*-polarized fundamental light). Such efficiency is also much larger than that from the 2D materials monolayer integrated with various resonance cavities even at the higher intensity of fundament light [19, 20, 24, 25]. Particularly, the non-dispersive flat bands in the whole moiré Brillouin zone of moiré superlattices can enable the high efficiency to be kept under the wide-angle illumination, which is superior to the other traditional resonance cavities of dispersive band structures or just local flat bands. The SHG field distributions at the two maximum peaks at the fundamental light of *x*- and *y*-polarization are shown in Figure 7(b) and (c), respectively. When the fundamental wavelength is fixed at the resonance mode, i.e. *P*_*x*_ or *P*_*y*_ mode, the dependence of SHG conversion efficiency on the intensity of fundamental light is shown in Figure 8. The slopes of the value 1 indicate the second-order nonlinear process. The derivation of 1 as the increase of the pump intensity of fundamental light is ascribed to the **P** ^(1)^(*ω*) due to the mixing of fundamental and SHG waves in MoS_2_ which shifts the resonance mode. Such large conversion efficiency in the 2D monolayers indicates the moiré superlattice is a promising platform for nonlinear photonics.

When the twist angle is reduced to be 6.01°, the resonance modes of moiré superlattice shift to 1456.709 nm (*P*_*x*_ mode) and 1456.715 nm (*P*_*y*_ mode) and the superlattice exhibits more confined field distribution. The maximum enhancement factor of local field is around 2.6 × 10^4^ (at the *P*_*x*_ mode) and 2.9 × 10^4^ (at the *P*_*y*_ mode) under the *x*- and *y*-polarization, respectively. The values are larger than those in moiré superlattices formed by the twist angle 9.43°. Under the low intensity of fundamental light 0.1 kW/cm^2^, the maximum SHG conversion efficiencies from a monolayer MoS_2_ are up to 6.5 × 10^−3^ (at the *P*_*x*_ mode) and 1.2 × 10^−2^ (at the *P*_*y*_ mode) at the illumination of *x*- and *y*-polarized fundamental light, respectively. It is noting that, besides the decrease of the twist angle for moiré superlattice to enhance the SHG, the further enhancement of SHG response depends on the optimization of nanostructures. The parameters of simple triangular lattices (the thickness of the slab, the radius of the holes, and the lattice constant) significantly affect the resonance modes even under the same twist angles. Experimentally, the limitation of the enhancement highly depends on the quality of materials and fabrications. Especially, the defects introduced during the nanofabrication and the surface roughness may dramatically decrease the *Q* factor due to the scattering and leaky processes to harm the SHG response.

Finally, we point out that the experimental verification of the numerical model is feasible. Nanostructured moiré superlattices formed by the triangular lattice of proper size of holes (radius 120 nm) and lattice constant (500 nm) can be fabricated by the state-of-the-art E-beam lithography and the etching processes, as reported in Ref. [34]. The wafer-scale monolayer MoS_2_ can be grown by chemical vapor deposition (CVD) method [46, 47] and can be transferred onto the desired substrates [46–49]. The linear optical transmission of the samples can be conducted using the supercontinuum generation laser as a light source and detectors (e.g. photodiode, photomultiplier tube, CCD). An optical parametric oscillator (OPO) femto-laser can be used for the measurement of SHG in the samples. The wavelength is tuned to the resonance states of the samples. Considering the laser of pulse repetition 80 MHz and pulse duration 200 fs, and the laser spot of 200 μm^2^ fully covering one unit of moire superlattice (9.43°, 8 μm^2^, 5.09°, 27.5 μm^2^, and 2.65°, 101.2 μm^2^), the average power of laser 1 μW can achieve the peak intensity 31.25 kW/cm^2^ for efficient SHG. The signals can be detected by the sensitive detectors, such as, photomultiplier tube, CCD, avalanche photodiode.

## 4 Conclusions

We investigate SHG response in monolayer 2D materials placed on the photonic moiré superlattice. The SHG conversion efficiency in 2D materials can be significantly enhanced via the greatly enhanced local field at the resonance modes of the moiré superlattice. For a typical TMD monolayer, such as MoS_2_, the maximum SHG conversion efficiency reaches up to 10^−1^ at an intensity of fundamental light 10 kW/cm^2^, which is about 14 orders of magnitude larger than that from the monolayer placed on a flat dielectric slab. Such nonlinear response from the monolayer can be further enhanced under the smaller twist angles of moiré superlattice. The flat bands in the whole moiré Brillouin zone enable the high efficiency from the nanostructure under the wide-angle illumination. Further, the intrinsic third-order optical nonlinearity of dielectrics is not considered for the simplification, which may shift the resonance frequency due to the change of refractive index and reduce the local field. However, the results still open a new possibility for enhancement of SHG in 2D-material monolayer based on photonic moiré superlattices.

## References

[1] Boyd R. W. (2008). *Nonlinear Optics*.

[2] Kumar N., Najmaei S., Cui Q. (2013). Second harmonic microscopy of monolayer MoS_2_. *Phys. Rev. B*.

[3] Malard L. M., Alencar T. V., Barboza A. P. M., Mak K. F., de Paula A. M. (2013). Observation of intense second harmonic generation from MoS_2_ atomic crystals. *Phys. Rev. B*.

[4] Seyler K. L., Schaibley J. R., Gong P. (2015). Electrical control of second-harmonic generation in a WSe_2_ monolayer transistor. *Nat. Nanotechnol.*.

[5] Jiang T., Huang D., Cheng J. (2018). Gate-tunable third-order nonlinear optical response of massless Dirac fermions in graphene. *Nat. Photonics*.

[6] Mennei L., Paur M., Mueller T. (2019). Second harmonic generation in strained transition metal dichalcogenide monolayers: MoS_2_, MoSe_2_, WS_2_, and WSe_2_. *APL Photonics*.

[7] Klimmer S., Ghaebi O., Gan Z. (2021). All-optical polarization and amplitude modulation of second-harmonic generation in atomically thin semiconductors. *Nat. Photonics*.

[8] Ferrari A. C., Bonaccorso F., Falko V. (2015). Science and technology roadmap for graphene, related two-dimensional crystals, and hybrid systems. *Nanoscale*.

[9] Liu Z., Liu J., Yin P. (2022). 2D Xenes: optical and optoelectronic properties and applications in photonic devices. *Adv. Funct. Mater.*.

[10] Yu S., Wu X., Wang Y., Guo X., Tong L. (2017). 2D materials for optical modulation: challenges and opportunities. *Adv. Mater.*.

[11] Cox J. D., Silveiro I., de Abajo F. G. (2016). Quantum effects in the nonlinear response of graphene plasmons. *ACS Nano*.

[12] Zhao Y., Huo Y., Man B., Ning T. (2019). Grating-assisted surface plasmon resonance for enhancement of optical harmonic generation in graphene. *Plasmonics*.

[13] Karimi F., Soleimanikahnoj S., Knezevic I. (2021). Tunable plasmon-enhanced second-order optical nonlinearity in transition metal dichalcogenide nanotriangles. *Phys. Rev. B*.

[14] Wang G., Marie X., Gerber I. (2015). Giant enhancement of the optical second-harmonic emission of WSe_2_ monolayers by laser excitation at exciton resonances. *Phys. Rev. Lett.*.

[15] Zeng J., Yuan M., Yuan W. (2015). Enhanced second harmonic generation of MoS_2_ layers on a thin gold film. *Nanoscale*.

[16] Shi J., Liang W.-Y., Raja S. S. (2018). Plasmonic enhancement and manipulation of optical nonlinearity in monolayer tungsten disulfide. *Laser Photonics Rev.*.

[17] Wang Z., Dong Z., Zhu H. (2018). Selectively plasmon-enhanced second-harmonic generation from monolayer tungsten diselenideon flexible substrates. *ACS Nano*.

[18] Fryett T. K., Seyler K. L., Zheng J., Liu C. H., Xu X., Majumdar A. (2017). Silicon photonic crystal cavity enhanced second-harmonic generation from monolayer WSe_2_. *2D Mater.*.

[19] Zhang Z., Zhang L., Gogna R., Chen Z., Deng H. (2020). Large enhancement of second harmonic generation in MoS_2_ by one dimensional photonic crystals. *Solid State Commun*..

[20] Khani M., Nezhad M. K., Rezaeiun H. R. M. (2018). Giant enhancement of second harmonic generation efficiency from MoS_2_ mono layers embedded in 1D photonic crystals. *Eur. Phys. J. Plus*.

[21] Chen H., Corboliou V., Solntsev A. S. (2017). Enhanced second harmonic generation from two-dimensional *MoSe*
_2_ on a silicon waveguide. *Light: Sci. Appl.*.

[22] Chen J. H., Tan J., Wu G. X., Zhang X. J., Xu F., Lu Y. Q. (2019). Tunable and enhanced light emission in hybrid WS_2_-optical-fiber-nanowire structures. *Light: Sci. Appl.*.

[23] Ngo G. O., Najafidehaghani E., Gan Z. (2022). In-fibre second harmonic generation with embedded two-dimensional materials. *Nat. Photonics*.

[24] Yi F., Ren M., Reed J. C. (2016). Optomechanical enhancement of doubly resonant 2D optical nonlinearity. *Nano Lett*..

[25] Day J. K., Chung M. H., Lee Y. H., Menon V. M. (2016). Microcavity enhanced second harmonic generation in 2D MoS_2_. *Opt. Mater. Express*.

[26] Shi J., Wu X., Zhang S. (2022). Giant enhancement and directional second harmonic emission from monolayer WS2 on silicon substrate via fabry-pérot micro-cavity. *ACS Nano*.

[27] Dean C. R., Wang L., Maher P. (2013). Hofstadter’s butterfly and the fractal quantum Hall effect in moiré superlattices. *Nature*.

[28] Cao Y., Fatemi V., Fang S. (2018). Unconventional superconductivity in magic-angle graphene superlattices. *Nature*.

[29] Cao Y., Fatemi V., Demir A. (2018). Correlated insulator behavior at half-filling in magic-angle graphene superlattices. *Nature*.

[30] Tran K., Moody G., Wu F. (2019). Evidence for moiré excitons in van der Waals heterostructures. *Nature*.

[31] Zhang W., Zou D., Pei Q., He W., Sun H., Zhang X. (2021). Moiré circuits: engineering magic-angle behavior. *Phys. Rev. B*.

[32] Wang P., Zheng Y., Chen X. (2020). Localization and delocalization of light in photonic moiré lattices. *Nature*.

[33] Fu Q., Wang P., Huang C. (2020). Optical soliton formation controlled by angle twisting in photonic moiré lattices. *Nat. Photonics*.

[34] Mao X. R., Shao Z. K., Luan H. Y., Wang S. L., Ma R.-M. (2021). Magic-angle lasers in nanostructured moiré superlattice. *Nat. Nanotechnol.*.

[35] Zhang Z., Liu D., Huo Y., Ning T. (2022). Ultralow-level all-optical self-switching in a nanostructured moiré superlattice. *Opt. Lett.*.

[36] Huang L., Zhang W., Zhang X. (2022). Moiré quasibound states in the continuum. *Phys. Rev. Lett.*.

[37] Zhang H. Z., Qin H. Y., Zhang W. X., Huang L., Zhang X. D. (2022). Moiré graphene nanoribbons: nearly perfect absorptions and highly efficient reflections with wide angles. *Opt. Express*.

[38] Ha S., Park N. H., Kim H. (2021). Enhanced third-harmonic generation by manipulating the twist angle of bilayer graphene. *Light: Sci. Appl.*.

[39] Yang F. Y., Song W. S., Meng F. H. (2020). Tunable second harmonic generation in twisted bilayer graphene. *Matter*.

[40] Du L. J., Dai Y. Y., Sun Z. P. (2020). Twisting for tunable nonlinear optics. *Matter*.

[41] Hong P., Xu L., Ying C., Rahmani M. (2022). Flatband mode in photonic moiré lattice for boosting second-harmonic generation with monolayer van der Waals crystals. *Opt. Lett.*.

[42] Ning T., Li X., Zhao Y. (2020). Giant enhancement of harmonic generation in all-dielectric resonant waveguide gratings of quasi-bound states in the continuum. *Opt. Express*.

[43] Islam K. M., Synowicki R., Ismael T., Oguntoye I., Grinalds N., Escarra M. D. (2021). In-plane and out-of-plane optical properties of monolayer, few-layer, and thin-film MoS_2_ from 190 to 1700 nm and their application in photonic device design. *Adv. Photonics Res.*.

[44] Li Y., Rao Y., Mak K. F. (2013). Probing symmetry properties of few-layer MoS_2_ and h-BN by optical second harmonic generation. *Nano Lett*..

[45] Wen X., Gong Z., Li D. (2019). Nonlinear optics of two-dimensional transition metal dichalcogenides. *InfoMat*.

[46] Yu H., Liao M., Zhao W. (2017). Wafer-scale growth and transfer of highly-oriented monolayer MoS_2_ continuous films. *ACS Nano*.

[47] Li T., Gao W., Ma L. (2021). Epitaxial growth of wafer-scale molybdenum disulfide semiconductor single crystals on sapphire. *Nat. Nanotechnol.*.

[48] Phan H. D., Kim Y., Lee J. (2017). Ultraclean and direct transfer of a wafer-scale MoS_2_ thin film onto a plastic substrate. *Adv. Mater.*.

[49] Sharma M., Singh A., Aggarwal P., Singh R. (2022). Large-area transfer of 2D TMDCs assisted by a water-soluble layer for potential device applications. *ACS Omega*.

